# Left Atrial Thrombus in Rheumatic Mitral Stenosis: A Case of Therapeutic International Normalized Ratio Failure

**DOI:** 10.1016/j.jaccas.2025.104884

**Published:** 2025-09-03

**Authors:** Muhammad Salman Sabri, Carly Weber, Yanis Belkadi, Donald Haas, Yasotha Rajeswaran

**Affiliations:** a Department of Internal Medicine, Jefferson Abington Hospital, Abington, Pennsylvania, USA; b Department of Cardiology, Lehigh Valley Hospital-Cedar Crest, Allentown, Pennsylvania, USA; c Department of Internal Medicine, Lehigh Valley Hospital-Cedar Crest, Allentown, Pennsylvania, USA

**Keywords:** atrial fibrillation, atrial flutter, cardioversion, left atrial appendage thrombus, rheumatic mitral stenosis, transesophageal echocardiogram

## Abstract

**Background:**

Atrial fibrillation or flutter in rheumatic mitral stenosis (MS) increases left atrial appendage (LAA) thrombus risk despite therapeutic anticoagulation.

**Case Summary:**

A 72-year-old woman on warfarin with moderate MS and atrial flutter presented with dyspnea. Transesophageal echocardiogram (TEE) showed a large LAA thrombus despite an international normalized ratio >2.5. Cardioversion was deferred, and the international normalized ratio target was raised to a range of 2.5 to 3.5.

**Take-Home Messages:**

While cardioversion can be considered in patients with uninterrupted anticoagulation without TEE, those with rheumatic MS are at high risk of LAA and LA thrombus and should undergo TEE before the procedure. Further research is needed to explore whether LAA thrombus warrants surgical intervention for rheumatic MS.

## Graphical Abstract

**Figure:**
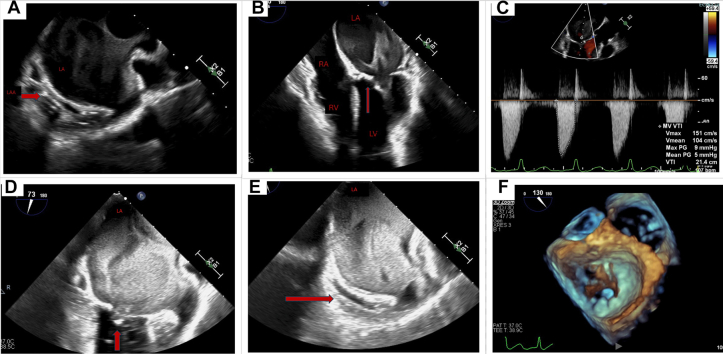

Regardless of the CHA_2_DS_2_-VASc score, patients with rheumatic mitral stenosis (MS) and atrial fibrillation (AF) or flutter are at high risk of thromboembolic events and require anticoagulation with vitamin K antagonists.^1^ While left atrial appendage (LAA) thrombus typically occurs with subtherapeutic international normalized ratio (INR), it can also develop in patients maintaining a therapeutic range.

**Take-Home Messages**

• Patients with rheumatic mitral stenosis remain at high risk of left atrial appendage thrombus despite therapeutic international normalized ratio on warfarin.
• Transesophageal echocardiogram should be considered before cardioversion in these patients, even with uninterrupted anticoagulation.
• The role of surgery in managing rheumatic mitral stenosis with persistent left atrial appendage thrombus requires further study.

## Case Presentation

A 72-year-old woman with rheumatic moderate MS, atrial flutter on sotalol and warfarin, and heart failure with preserved ejection fraction presented with worsening dyspnea. She had a consistent therapeutic INR (>2.5) for over a year. On arrival, her vitals showed a heart rate of 170 beats/min, mean arterial pressure of 70 mm Hg, and oxygen saturation of 84% on 4L oxygen. Physical exam revealed signs of volume overload and a diastolic murmur. Electrocardiogram showed atrial flutter at 153 BPM. Labs noted an INR of 2.8, B-type natriuretic peptide of 410 pg/mL, and mildly elevated troponin. Chest x-ray showed interstitial edema.

**Figure 1:**
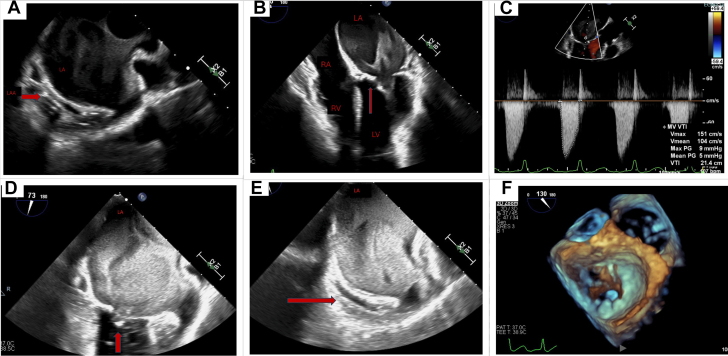
Transesophageal Echocardiogram The transesophageal echocardiogram (TEE) reveals smoke in the left atrium (LA) and a thrombus in the left atrial appendage (LAA), indicated by the arrow, which occupies nearly the entire body of the LAA (A). The mitral valve appears calcified and stenotic, as shown by the arrows in B and D, and there is a 5-mm Hg gradient across mitral valve consistent with moderate mitral stenosis (C). Definity contrast images illustrate the filling defect in the LAA indicating an LAA thrombus (D and E). (F) A 3D picture of the stenotic mitral valve. LV = left ventricle; RA = right artery; RV = right ventricle.

She underwent transesophageal echocardiogram (TEE) before planned cardioversion. TEE (Figure 1, Videos 1 to 4) revealed a normal left ventricle with preserved systolic function but a dilated left atrium with a large, fixed thrombus completely occluding the LAA and reduced LAA velocity (<40 cm/s), consistent with impaired function and high thrombotic risk. Progressive MS was confirmed, with a mean gradient of 5 mm Hg (heart rate: 107 beats/min) and mitral valve area of 1.5 cm^2^. Due to the thrombus, cardioversion was deferred. Her INR target was increased to a range of 2.5 to 3.5, sotalol was stopped, and repeat TEE was scheduled in 3 months.

## Discussion

Warfarin remains the anticoagulant of choice in rheumatic MS with AF or flutter,^1^ as direct oral anticoagulants have not been adequately studied in this group.^2^ However, as this case highlights, therapeutic INR does not eliminate thrombus risk due to persistent blood stasis and structural abnormalities in the left atrium and LAA.

Current guidelines allow for cardioversion without TEE in patients on uninterrupted anticoagulation for 3 or more weeks.^3^ However, this approach may not be appropriate in rheumatic MS, where the thrombotic risk remains elevated despite anticoagulation. Our case illustrates the need to individualize care—TEE should be strongly considered prior to cardioversion in rheumatic MS, even if the patient has been consistently anticoagulated.

The presence of a thrombus despite therapeutic INR raises questions about optimal INR targets, which may need to be higher (2.5-3.5) in this population. The use of aspirin in addition to oral anticoagulation may be considered to reduce the risk of thromboembolic events in patients at high risk with chronic AF.^4^ In our case, aspirin was added in addition to increasing INR goal given the dense spontaneous echo contrast in the LA and potential thrombus in LAA. In addition, the role of surgery in rheumatic MS complicated by LAA thrombus warrants further investigation. Valve surgery may reduce LA stasis and future thrombus risk, potentially improving long-term outcomes.

## Funding Support and Author Disclosures

The authors have reported that they have no relationships relevant to the contents of this paper to disclose.

## Appendix

Video 1 Transesophageal Echocardiogram Showing Smoke in the Left Atrium and Stenotic Calcified Mitral Valve With Mitral Annular Calcification

Video 2 Transesophageal Echocardiogram Demonstrates Swirling of Contrast in the Left Atrium Concerning Stasis of Blood Flow in the Left Atrium and Stenotic Mitral Valve

Video 3 Transesophageal Echocardiogram Demonstrates Thrombus in the Left Atrial Appendage

Video 4 Transesophageal Echocardiogram 3D Demonstrated Mitral Stenosis of Moderate Severity With a Mitral Valve Area of 1.5 cm^2^
